# Supplementary material for: Toll-like receptor signaling in thymic epithelium controls monocyte-derived dendritic cell recruitment and Treg generation
Source: Nat Commun. 2020 May 12;11:2361. doi: 10.1038/s41467-020-16081-3 (PMC7217920; doi:10.1038/s41467-020-16081-3)
Supplement: Supplementary file 1 — Supplementary Information [file 41467_2020_16081_MOESM1_ESM.pdf]

Supplementary information

Toll-like receptor signaling in thymic epithelium controls monocyte-derived dendritic cell recruitment and Treg generation

Matouš Vobořil, Tomáš Brabec, Jan Dobeš, Iva Šplíchalová, Jiří Březin, Adéla Čepková, Martina Dobešová, Aigerim Aidarova, Jan Kubovčiak, Oksana Tsyklauri, Ondřej Štěpánek, Vladimír Beneš, Radislav Sedláček, Ludger Klein, Michal Kolář and Dominik Filipp

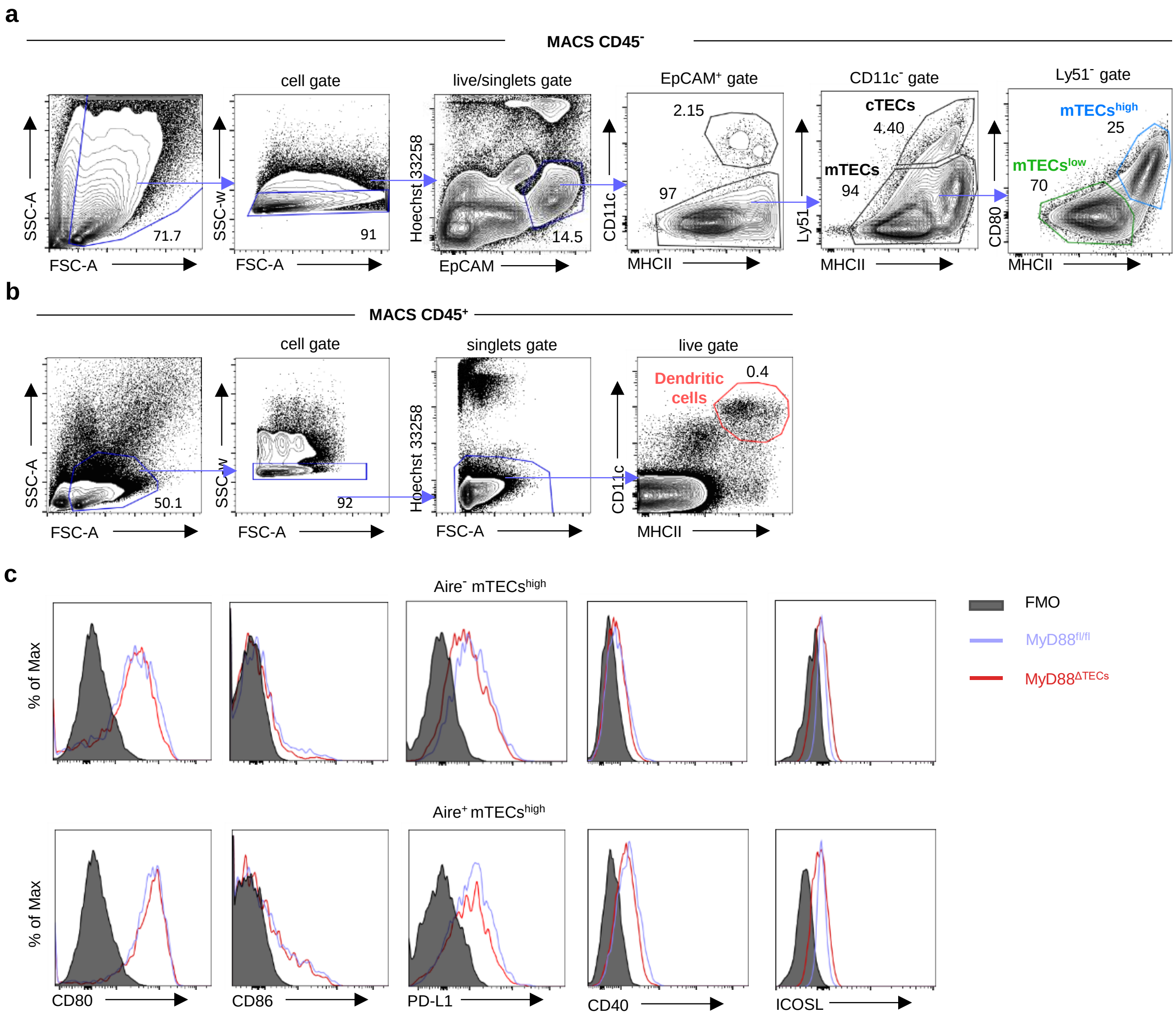

Supplementary Fig. 1, Related to Fig. 1 | Gating strategies and MyD88 independent expression of co-stimulatory molecules by mTECs. a, b Complete gating strategies of TECs and thymic classical DCs related to Fig. 1a. Thymic cell fraction were MACS depleted according to the expression of CD45. CD45⁻ fraction was sequentially gated according to FSC-A and SSC-A, singlets, live EpCAM⁺ and CD11c⁻ cells. The EpCAM⁺CD11c⁻ fraction representing TECs was then gated as cTECs (Ly51⁺) and mTECs (Ly51⁻). mTECs consist of two major populations: mTECs^{low} (MHCII^{low}CD80^{low}) and mTECs^{high} (MHCII^{high}CD80^{high}). CD45⁺ fraction was gated according to the FSC-A, SSC-A, SSC-W and Hoechst 33258 to live, single cells. Thymic conventional DCs were gated as MHCII⁺CD11c^{high}. **c** Representative flow cytometry histograms of co-stimulatory molecules (CD80, CD86, PD-L1, CD40 and ICOSL) expression on Aire⁻ mTECs^{high} and Aire⁺ mTECs^{high} of MyD88$^{fl/fl}$ and MyD88$^{\Delta TECs}$ mice, *n*=2 independent experiments.

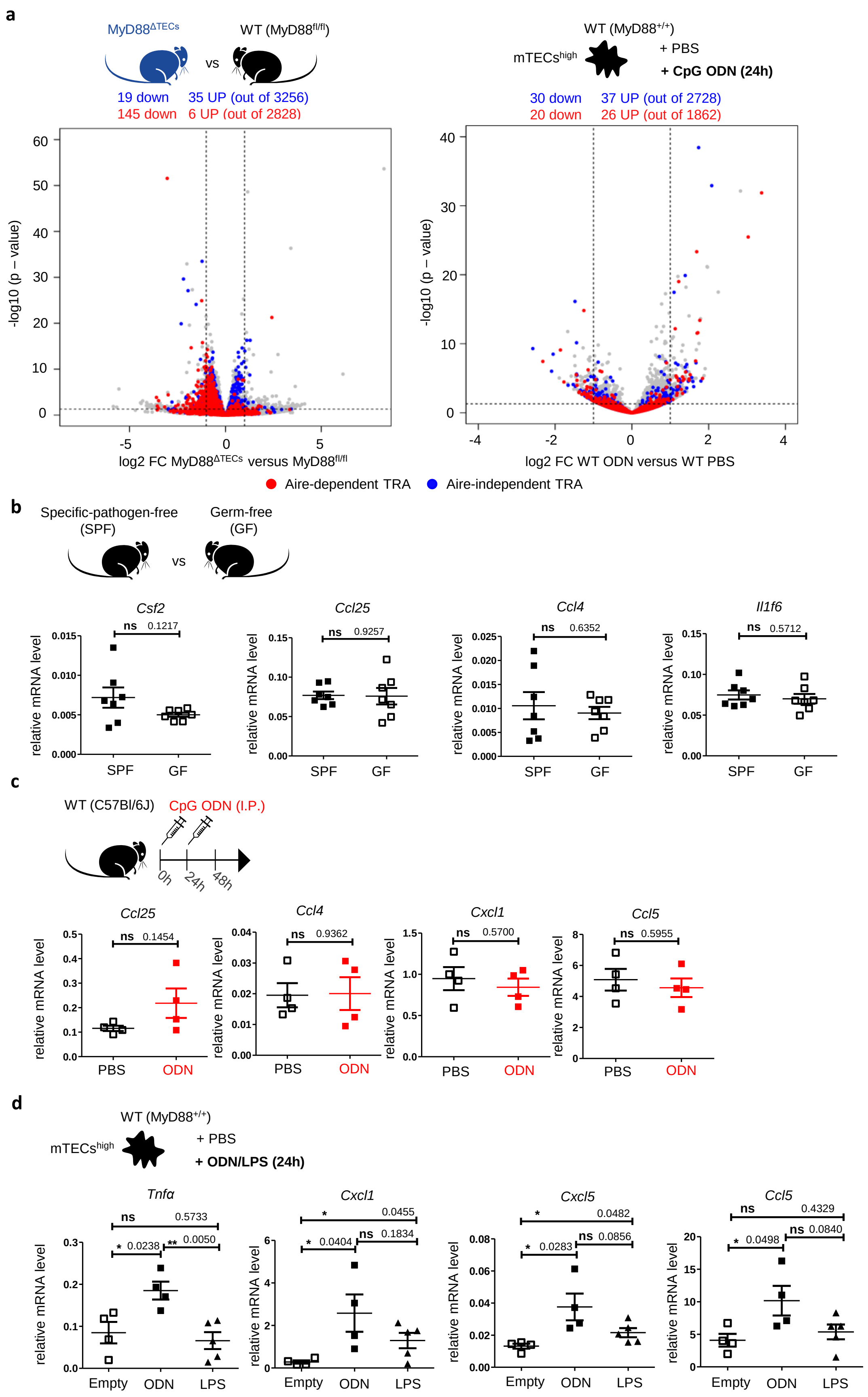

a
MyD88ΔTECs
vs
WT (MyD88fl/fl)
19 down
35 UP (out of 3256)
145 down
6 UP (out of 2828)
-log10 (p – value)
log2 FC MyD88ΔTECs versus MyD88fl/fl
WT (MyD88+/+)
mTECshigh
+ PBS
+ CpG ODN (24h)
30 down
37 UP (out of 2728)
20 down
26 UP (out of 1862)
log2 FC WT ODN versus WT PBS
Aire-dependent TRA
Aire-independent TRA
b
Specific-pathogen-free (SPF)
Germ-free (GF)
vs
Csf2
ns 0.1217
Ccl25
ns 0.9257
Ccl4
ns 0.6352
Il1f6
ns 0.5712
relative mRNA level
SPF
GF
c
WT (C57Bl/6J)
CpG ODN (I.P.)
0h
24h
48h
Ccl25
ns 0.1454
Ccl4
ns 0.9362
Cxcl1
ns 0.5700
Ccl5
ns 0.5955
PBS
ODN
d
WT (MyD88+/+)
mTECshigh
+ PBS
+ ODN/LPS (24h)
Tnfα
ns 0.5733
* 0.0238
** 0.0050
Cxcl1
* 0.0455
* 0.0404
ns 0.1834
Cxcl5
* 0.0482
* 0.0283
ns 0.0856
Ccl5
ns 0.4329
* 0.0498
ns 0.0840
Empty
ODN
LPS

Supplementary Fig. 2, **Related to Fig. 2 | TLR/MyD88 signaling in mTECs^{high} drives the expression of cytokines and chemokine independently on presence of microbiota. a** Volcano plot analysis of RNA-sequencing data from Fig. 2b (left plot) and 2e (right plot) showing comparison of Aire-dependent (red) and Aire-independent (blue) TRA genes, non TRA genes are in grey. Statistical analysis was performed by Wald test, Fold-change cutoff of log2=+/-1,0 and p-value: 0,05 are marked by dashed lines (*n*=5 samples for left plot and *n*=4 samples for right plot). **b** qRT-PCR analysis of mRNA expression (normalized to Cacs3) of genes selected based on the Fig. 2b from mTEC^{high} isolated from Specific-pathogen-free (SPF) or Germ-free (GF) WT (C57Bl/6J) mice (mean ± SEM, *n*=7 mice). **c** qRT-PCR analysis of mRNA expression (normalized to Cacs3) of genes selected based on the Fig. 2b and e from intraperitoneally CpG ODN stimulated WT (C57Bl/6J) mice (mean ± SEM, *n*=4 mice). **d** qRT-PCR analysis of mRNA expression (normalized to Cacs3) of genes selected based on the Fig. 2e from *in vitro* CpG ODN or LPS stimulated mTECs^{high} (mean ± SEM, *n*=4 for Empty and ODN and *n*=5 for LPS stimulated samples). Statistical analysis in b, c and d was performed by unpaired, two-tailed Student's t-test, $p \leq 0.05$ = *, $p \leq 0.01$ = **, ns = not significant.

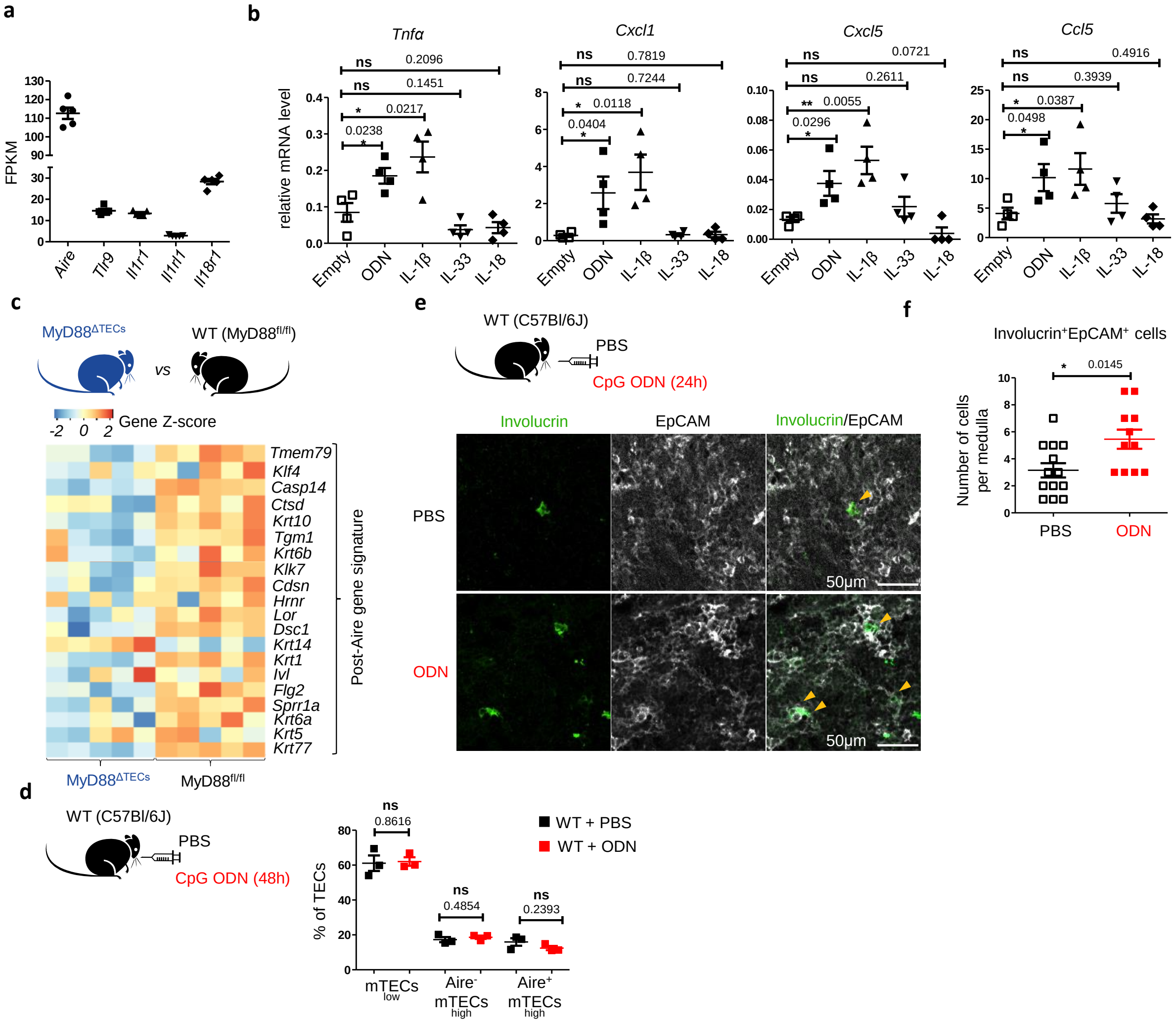

Supplementary Fig. 3, **Related to Fig. 2 | TLR/MyD88 signaling affects the development of post-Aire mTECs. a** Expression of indicated genes in mTECs^{high} depicted as FPKM (Fragments normalized per kilobase of feature lenght per million mapped fragments) (mean ± SEM, *n*=5 samples). **b** qRT-PCR analysis of mRNA expression (normalized to Cacs3) of genes selected based on the Fig. 2e from *in vitro* CpG ODN, IL-1β, IL-33 and IL-18 stimulated mTECs^{high} (mean ± SEM, *n*=4 samples). **c** Comparison of the expression of signature genes associated with post-Aire mTECs between MyD88$^{\Delta TECs}$ and MyD88$^{fl/fl}$ mice. Data are derived from RNAseq analysis shown in Fig. 2b. **d** The frequencies of mTECs (gated as in Fig. 1e) isolated from CpG ODN or PBS intrathymically stimulated WT (C57Bl/6J) mice and analyzed by flow cytometry (mean ± SEM, *n*=3 mice). **e** Microscopic examinations of thymic sections isolated from CpG ODN or PBS intrathymically stimulated WT (C57Bl/6J) mice. Cryosections were stained with Involucrin (green) and EpCAM (white). Scale bar represents 50μm. **f** Enumeration of Involucrin^{+}EpCAM^{+} cells in the medullar region of the cryosections shown in e (mean ± SEM, *n*=11 for ODN and *n*=13 for PBS stimulated medullary regions from 3 independent experiments). Statistical analysis in b, d and f was performed by unpaired, two-tailed Student's t-test, p ≤ 0.05 = *, p ≤ 0.01 = **, ns = not significant.

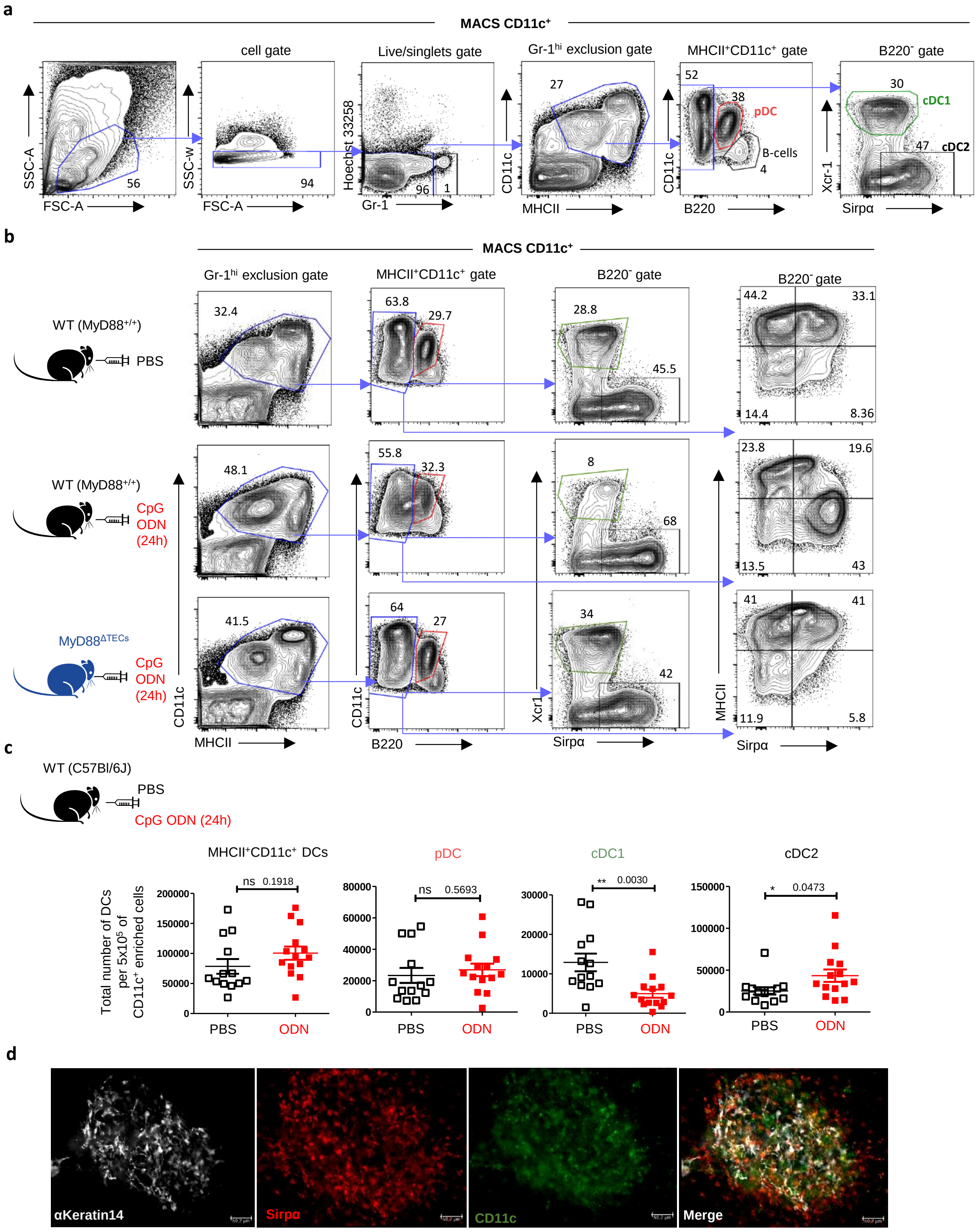
a
MACS CD11c+
cell gate
Live/singlets gate
Gr-1hi exclusion gate
MHCII+CD11c+ gate
B220- gate
SSC-A
FSC-A
56
SSC-w
FSC-A
94
Hoechst 33258
Gr-1
96
1
27
CD11c
MHCII
52
38
pDC
B-cells
4
CD11c
B220
30
cDC1
47
cDC2
Xcr-1
Sirpα
b
MACS CD11c+
Gr-1hi exclusion gate
MHCII+CD11c+ gate
B220- gate
B220- gate
WT (MyD88+/+)
PBS
32.4
63.8
29.7
28.8
45.5
44.2
33.1
14.4
8.36
WT (MyD88+/+)
CpG
ODN
(24h)
48.1
55.8
32.3
8
68
23.8
19.6
13.5
43
MyD88ΔTECs
CpG
ODN
(24h)
41.5
64
27
34
42
41
41
11.9
5.8
CD11c
MHCII
CD11c
B220
Xcr1
Sirpα
MHCII
Sirpα
c
WT (C57Bl/6J)
PBS
CpG ODN (24h)
MHCII+CD11c+ DCs
pDC
cDC1
cDC2
Total number of DCs
per 5x10^5 of
CD11c+ enriched cells
200000
150000
100000
50000
0
ns 0.1918
PBS
ODN
80000
60000
40000
20000
0
ns 0.5693
PBS
ODN
30000
20000
10000
0
** 0.0030
PBS
ODN
150000
100000
50000
0
* 0.0473
PBS
ODN
d
αKeratin14
Sirpα
CD11c
Merge

Supplementary Fig. 4, Related to Fig. 3 | Representative gating strategies and localization of thymic DCs. a Complete gating strategy of thymic DCs subpopulations. Thymic cells were MACS enriched for $CD11c^{+}$ cells and sequentially gated according to FSC-A and SSC-A, singlets and live cells. Total thymic DCs were gated as $Gr\text{-}1^{-}CD11c^{+}MHCII^{+}$. Thymic DCs were then divided into three major populations; pDC = $CD11c^{low}MHCII^{low}B220^{+}$, cDC1 = $CD11c^{+}MHCII^{+}B220^{-}Xcr1^{+}Sirp\alpha^{-}$ and cDC2 = $CD11c^{+}MHCII^{+}B220^{-}Xcr1^{-}Sirp\alpha^{+}$. **b** Representative flow cytometry plots showing frequency of thymic DC subpopulations in CpG ODN or PBS intrathymically stimulated $MyD88^{fl/fl}$ or $MyD88^{\Delta TECs}$ mice. **c** Quantification of the total number of thymic DCs, isolated as $CD11c^{+}$ MACS enriched population from CpG ODN or PBS intrathymically stimulated WT (C57Bl/6J) mice (mean ± SEM, *n*=13 for PBS and *n*=14 for ODN stimulated mice)**.** Statistical analysis was performed by unpaired, two-tailed Student's t-test, $p \leq 0.05$ = *, $p \leq 0.01$ = **, ns = not significant. **d** Microscopic analysis of the localization of $Sirp\alpha^{+}$mDCs in WT (C57Bl/6J) thymic medullary region determined by Keratin-14 staining. Scale bar represents 50.2 μm (*n*=3 independent experiments).

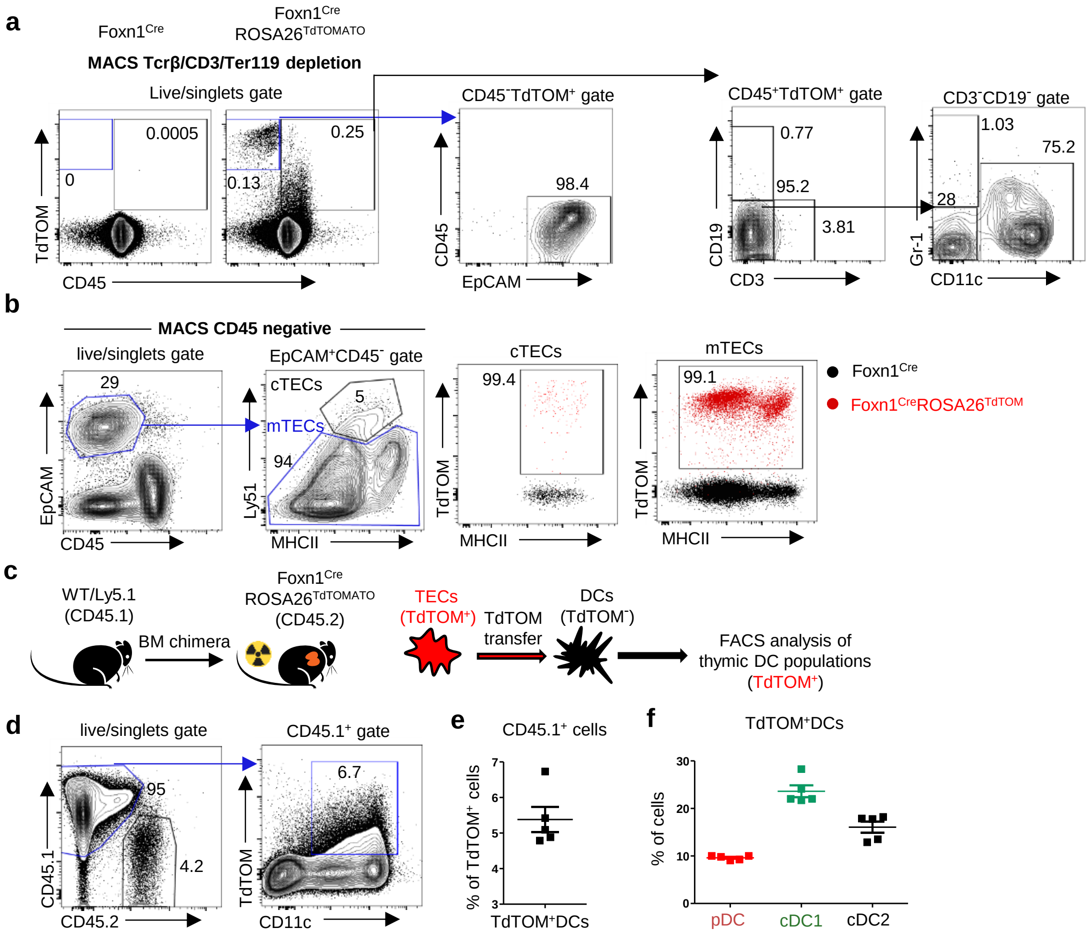

Supplementary Fig. 5, Related to Fig. 4 | Foxn1^{Cre}ROSA26^{TdTOMATO} as a model of thymic cooperative antigen transfer. a Representative flow cytometry plots showing frequency of TdTOM^{+} cells among MACS TCRβ, CD3 and Ter119 depleted thymic cells from WT (Foxn1^{Cre}) and Foxn1^{Cre}ROSA26^{TdTOMATO} mice (*n*=3 independent experiments). CD45^{+}TdTOM^{+} cells were further divided by CD19, CD3, Gr-1 and CD11c protein staining. **b** Flow cytometry analysis of TdTOM expression in CD45^{-}depleted and EpCAM^{+} thymic epithelial populations. The level of TdTOM is projected as an overlay of gated subset (cTECs or mTECs, right panels) from Foxn1^{Cre} (black dots) and Foxn1^{Cre}ROSA26^{TdTOMATO} (red dots) mice (*n*=3 independent experiments). The number indicates the percentage of TdTOM^{+} cells from gated subset. **c** Design of experiment. **d** Representative flow cytometry plots showing the frequency of TdTOM^{+}CD11c^{+} cells in the thymic MACS-enriched CD11c donor cells from WT (CD45.1^{+}) to Foxn1^{Cre}ROSA26^{TdTOMATO} BM chimeras. **e** Quantification of the frequencies of CD45.1^{+}TdTOM^{+} DCs (mean ± SEM, *n*=5 mice). **f** Quantification of frequencies of CD45.1^{+}TdTOM^{+} DCs among DC subsets (gated as in Supplementary Fig. 3a) (mean ± SEM, *n*=5 mice).

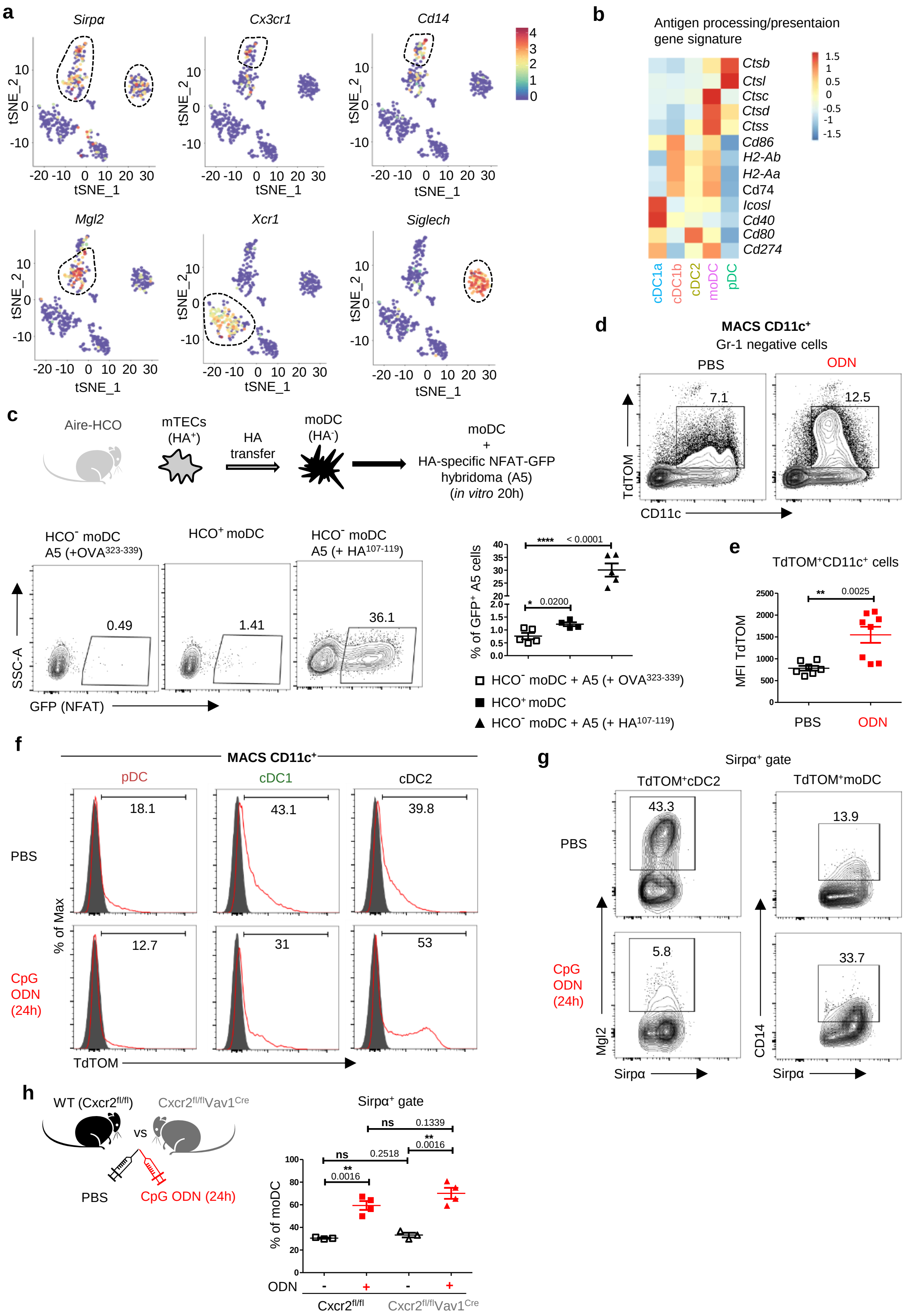
a
Sirpα
Cx3cr1
Cd14
Mgl2
Xcr1
Siglech
tSNE_1
tSNE_2
b
Antigen processing/presentaion gene signature
Ctsb
Ctsl
Ctsc
Ctsd
Ctss
Cd86
H2-Ab
H2-Aa
Cd74
Icosl
Cd40
Cd80
Cd274
cDC1a
cDC1b
cDC2
moDC
pDC
c
Aire-HCO
mTECs (HA+)
HA transfer
moDC (HA-)
moDC + HA-specific NFAT-GFP hybridoma (A5) (in vitro 20h)
HCO- moDC A5 (+OVA323-339)
HCO+ moDC
HCO- moDC A5 (+ HA107-119)
0.49
1.41
36.1
SSC-A
GFP (NFAT)
% of GFP+ A5 cells
< 0.0001
0.0200
HCO- moDC + A5 (+ OVA323-339)
HCO+ moDC
HCO- moDC + A5 (+ HA107-119)
d
MACS CD11c+
Gr-1 negative cells
PBS
ODN
7.1
12.5
TdTOM
CD11c
e
TdTOM+CD11c+ cells
0.0025
MFI TdTOM
PBS
ODN
f
MACS CD11c+
pDC
cDC1
cDC2
PBS
CpG ODN (24h)
18.1
43.1
39.8
12.7
31
53
% of Max
TdTOM
g
Sirpα+ gate
TdTOM+cDC2
TdTOM+moDC
PBS
CpG ODN (24h)
43.3
13.9
5.8
33.7
Mgl2
CD14
Sirpα
h
WT (Cxcr2fl/fl)
vs
Cxcr2fl/flVav1Cre
PBS
CpG ODN (24h)
Sirpα+ gate
ns
0.1339
0.2518
0.0016
0.0016
% of moDC
ODN
Cxcr2fl/fl
Cxcr2fl/flVav1Cre

Supplementary Fig. 6, Related to Fig. 5 | TLR/MyD88 signaling increases cooperative antigen transfer between TECs and CD14^{+} moDC subpopulation. a Heat-map projection of selected signature genes (based on the Fig. 5a) between individual clusters (dashed circles) onto the two-dimensional tSNE plot. **b** Heat-map analysis of the expression of antigen processing/presentation signature genes in each subset defined in Fig. 5a. **c** Indirect presentation of Influenza Haemaglutinin (HA) by CD14^{+}moDC (sorted as CD11c^{+}MHCII^{+}B220^{-}Xcr1^{-}Cx3cr1^{+}CD14^{+}) using Aire-HCO mice model and A5 hybridoma T-cell line. Representative flow cytometry plots showing frequency of GFP^{+} A5 cells co-cultivates with moDCs sorted form Aire-HCO^{-} (pulsed with irrelevant OVA peptide, left plot), Aire-HCO^{+} mice (middle plot) and Aire-HCO^{-} (pulsed with HA peptide, right plot). The graph shows the quantification of frequency of GFP^{+} A5 cells (mean ± SEM, *n*=4 for HCO^{+}moDC and *n*=5 for HCO^{-}moDC + A5 independent experiments). **d** Representative flow cytometry plots comparing the frequency of Gr-1^{-}TdTOM^{+}CD11c^{+} cells between CpG ODN and PBS intrathymically stimulated Foxn1^{Cre}ROSA26^{TdTOMATO} mice. **e** Quantification of TdTOMATO Mean fluorescent intensity (MFI) in mice described in d (mean ± SEM, *n*=7 for PBS and *n*=8 for ODN mice). **f** Representative flow cytometry plots showing frequency of TdTOM^{+} cells among pDCs, cDC1 and cDC2 (gated as in Supplementary Fig. 4a) in mice described in Fig. 5d. Grey histograms represent control Foxn1^{Cre} mice. **g** Representative flow cytometry plots showing frequency of Mgl2^{+}cDC2 or CD14^{+}moDC cells among TdTOM^{+}Sirpα^{+} DCs in CpG ODN or PBS intrathymically stimulated Foxn1^{Cre}ROSA26^{TdTOMATO} mice. **h** Quantification of frequencies of TdTOM^{+}CD14^{+} moDC from CpG ODN or PBS intrathymically stimulated Cxcr2$^{fl/fl}$ or Cxcr2$^{fl/fl}$Vav1Cre mice (mean ± SEM, *n*=3 for ODN^{-} and *n*=4 for ODN^{+} mice). Statistical analysis in c, e and h was performed by unpaired, two-tailed Student's t-test, $p \leq 0.05$ = *, $p \leq 0.01$ = **, $p < 0.0001$ = ****, ns = not significant.

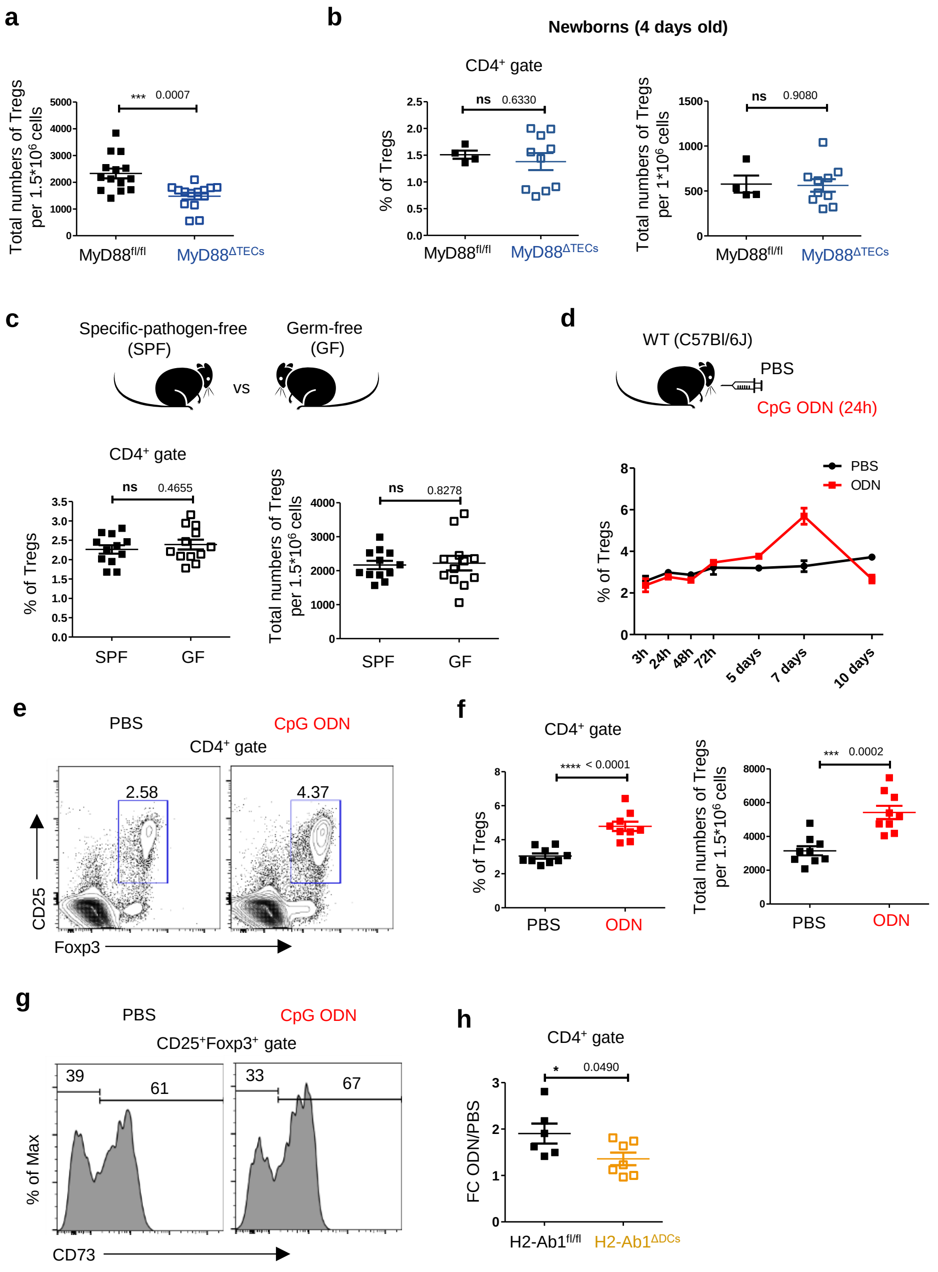

a
Total numbers of Tregs per 1.5*10^6 cells
*** 0.0007
MyD88fl/fl
MyD88ΔTECs
b
Newborns (4 days old)
CD4+ gate
% of Tregs
ns 0.6330
Total numbers of Tregs per 1*10^6 cells
ns 0.9080
MyD88fl/fl
MyD88ΔTECs
c
Specific-pathogen-free (SPF)
vs
Germ-free (GF)
CD4+ gate
% of Tregs
ns 0.4655
Total numbers of Tregs per 1.5*10^6 cells
ns 0.8278
SPF
GF
d
WT (C57Bl/6J)
PBS
CpG ODN (24h)
PBS
ODN
% of Tregs
3h
24h
48h
72h
5 days
7 days
10 days
e
PBS
CpG ODN
CD4+ gate
2.58
4.37
CD25
Foxp3
f
CD4+ gate
% of Tregs
**** < 0.0001
Total numbers of Tregs per 1.5*10^6 cells
*** 0.0002
PBS
ODN
g
PBS
CpG ODN
CD25+Foxp3+ gate
39
61
33
67
% of Max
CD73
h
CD4+ gate
FC ODN/PBS
* 0.0490
H2-Ab1fl/fl
H2-Ab1ΔDCs

Supplementary Fig. 7, Related to Fig. 6 | Development of $CD25^+Foxp3^+$ thymic Tregs is impaired in the absence of TLR/MyD88 signaling in TECs. a Quantification of the total number of $CD4^+CD25^+Foxp3^+$ thymic Tregs in $MyD88^{fl/fl}$ and $MyD88^{\Delta TECs}$ (mean ± SEM, *n*=14 mice). **b** Quantification of frequencies (left graph) and total numbers (right plot) of $CD4^+CD25^+Foxp3^+$ thymic Tregs isolated form the newborn (4 days old) $MyD88^{fl/fl}$ and $MyD88^{\Delta TECs}$ mice (mean ± SEM, *n*=4 for $MyD88^{fl/fl}$ and *n*=10 for $MyD88^{\Delta TECs}$ mice). **c** Quantification of frequencies (left graph) and total numbers (right plot) of $CD4^+CD25^+Foxp3^+$ thymic Tregs in Specific-pathogen-free (SPF) and Germ-free (GF) WT (C57Bl/6J) mice (mean ± SEM, *n*=12 mice). **d** Quantification of the frequencies of $CD4^+CD25^+Foxp3^+$ thymic Tregs from CpG ODN or PBS intrathymically stimulated WT (C57Bl/6J) mice over the indicated period of time, from 3 hours to 10 days (mean ± SEM, *n*=2 for 48h, 72h and 10days PBS and 10days ODN and *n*=3 for other displayed items). **e** Representative flow cytometry plots comparing frequencies of $CD4^+CD25^+Foxp3^+$ thymic Tregs in CpG ODN or PBS intrathymically stimulated WT (C57Bl/6J) mice 7 days after stimulation. **f** Quantification of frequencies and the total numbers of thymic Tregs from mice described in e, (mean ± SEM, *n*=9 mice). **g** Representative flow cytometry histograms showing the expression of CD73 by $CD4^+CD25^+Foxp3^+$ thymic Tregs in CpG ODN or PBS intrathymically stimulated WT (C57Bl/6J) mice. **h** Fold-change increase in the cellularity of thymic Tregs in $H2\text{-}Ab1^{fl/fl}$ and $H2\text{-}Ab1^{\Delta DCs}$ mice stimulated with CpG ODN (mean ± SEM, *n*=6 for $H2\text{-}Ab1^{fl/fl}$ and *n*=7 for $H2\text{-}Ab1^{\Delta DCs}$ mice). Data were calculated from Fig. 6h. Statistical analysis in a, b, c, f and h was performed by unpaired, two-tailed Student's t-test, $p \leq 0.05$ = *, $p \leq 0.001$ = ***, $p < 0.0001$ = ****, ns = not significant.

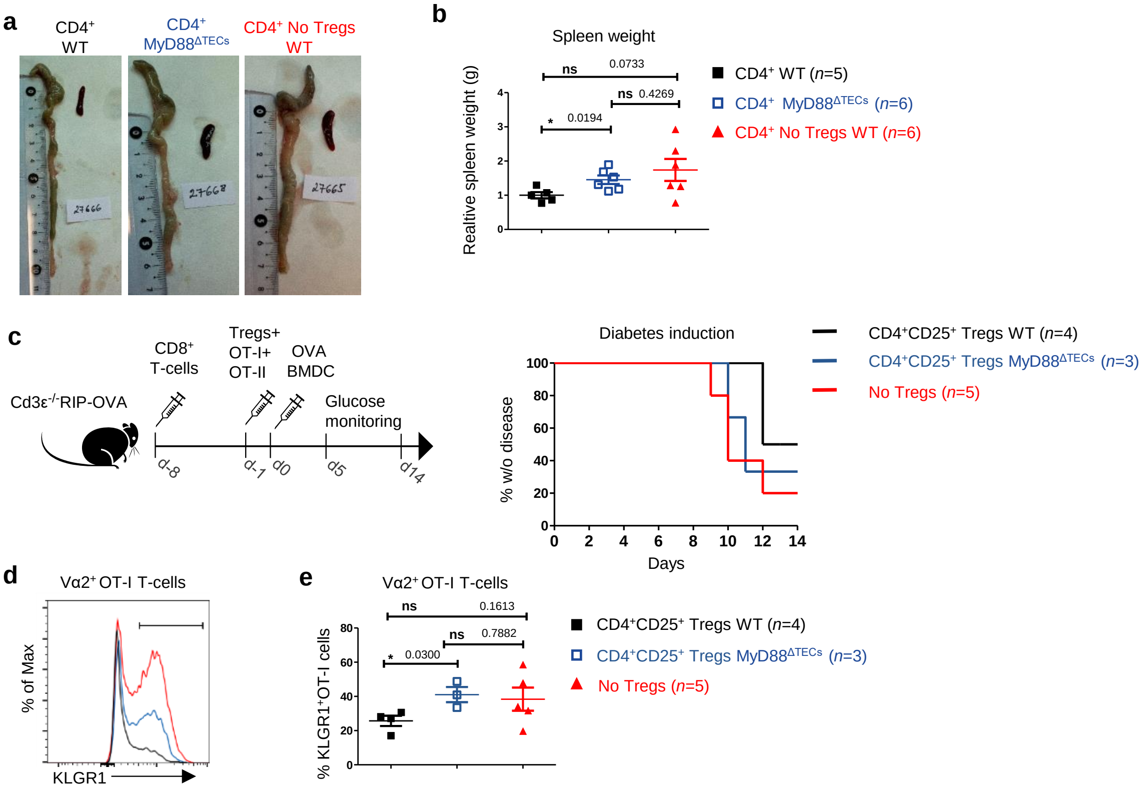

Supplementary Fig. 8, Related to Fig. 7 | Tregs cells from MyD88$^{\Delta TECs}$ mice have reduced suppressive capacity and failed to prevent the development of T-cell induced colitis or diabetes. a Representative pictures showing the size of colon and spleen from indicated mice (described in Figure 7a). **b** Relative quantification (normalized to average of control mice from each experiment) of spleen weight of T-cell induced colitis experimental mice (mean ± SEM, *n*=5-6 mice). Statistical analysis was performed by unpaired, two-tailed Student's t-test, $p \leq 0.05$ = *, ns = not significant. **c** Design of experiment and disease progression curve showing the percentage of non-diabetic mice. Number of *n* for each cohort is indicated. **d** Representative flow cytometry histograms of KLGR1 expression in Vα2^{+}OT-I T-cells isolated at day 14 from spleen of mice from c. **e** Quantification of frequencies of KLGR1^{+}OT-I T-cells from d (mean ± SEM, *n*=3-5 mice). Statistical analysis was performed by unpaired, two-tailed Student's t-test, $p \leq 0.05$ = *, ns = not significant.
