# Supplementary material for: Toll-like receptor signaling in thymic epithelium controls monocyte-derived dendritic cell recruitment and Treg generation
Source: Nat Commun. 2020 May 12;11:2361. doi: 10.1038/s41467-020-16081-3 (PMC7217920; doi:10.1038/s41467-020-16081-3)
Supplement: Supplementary file 3 — Reporting Summary [file 41467_2020_16081_MOESM3_ESM.pdf]

nature research

Corresponding author(s): Dominik Filipp

Last updated by author(s): Mar 25, 2020

Reporting Summary

Nature Research wishes to improve the reproducibility of the work that we publish. This form provides structure for consistency and transparency in reporting. For further information on Nature Research policies, see Authors & Referees and the Editorial Policy Checklist.

Statistics

For all statistical analyses, confirm that the following items are present in the figure legend, table legend, main text, or Methods section.

n/a	Confirmed	
☐	☒	The exact sample size (*n*) for each experimental group/condition, given as a discrete number and unit of measurement
☐	☒	A statement on whether measurements were taken from distinct samples or whether the same sample was measured repeatedly
☐	☒	The statistical test(s) used AND whether they are one- or two-sided<br>*Only common tests should be described solely by name; describe more complex techniques in the Methods section.*
☒	☐	A description of all covariates tested
☐	☒	A description of any assumptions or corrections, such as tests of normality and adjustment for multiple comparisons
☐	☒	A full description of the statistical parameters including central tendency (e.g. means) or other basic estimates (e.g. regression coefficient) AND variation (e.g. standard deviation) or associated estimates of uncertainty (e.g. confidence intervals)
☒	☐	For null hypothesis testing, the test statistic (e.g. *F*, *t*, *r*) with confidence intervals, effect sizes, degrees of freedom and *P* value noted<br>*Give P values as exact values whenever suitable.*
☒	☐	For Bayesian analysis, information on the choice of priors and Markov chain Monte Carlo settings
☒	☐	For hierarchical and complex designs, identification of the appropriate level for tests and full reporting of outcomes
☒	☐	Estimates of effect sizes (e.g. Cohen's *d*, Pearson's *r*), indicating how they were calculated

Our web collection on statistics for biologists contains articles on many of the points above.

Software and code

Policy information about availability of computer code

Data collection	BD FACSDIVA™ SOFTWARE V6.0 for LSRII (with HTS option), Roche LightCycler 480 1.5 software, IDEAS 6.1 software
Data analysis	GraphPad Prism 5.04, FlowJo V10 (10.5.3), Roche LightCycler 480 1.5 software, ImageJ (version Figi64), SciLifeLab/NGI-RNAseq R Package (https://github.com/SciLifeLab/NGI-RNAseq), DESeq2 R Bioconductor (version 3.10), FastQC (version 0.11.9), Seurat R Package (version 3.0), UMI-tools, HISAT2 (version 2.1.0), feautureCounts (version 2.0.0), Trim Galore! (version 0.4.5)

For manuscripts utilizing custom algorithms or software that are central to the research but not yet described in published literature, software must be made available to editors/reviewers. We strongly encourage code deposition in a community repository (e.g. GitHub). See the Nature Research guidelines for submitting code & software for further information.

Data

Policy information about availability of data

All manuscripts must include a data availability statement. This statement should provide the following information, where applicable:

- Accession codes, unique identifiers, or web links for publicly available datasets
- A list of figures that have associated raw data
- A description of any restrictions on data availability

The authors declare that all data supporting the findings of this study are available within the article and its supplementary information files or from the corresponding author upon reasonable request. The source data underlying Figs. 1c, f, 2c, f, g, 3a-c, e, 4d, f, 5d, f, g, 6a, c, e-i, 7b, d-h and Supplementary Figs 2b-d, 3a, b, d, e, 4c, 5e, f, 6c, e, h, 7a-d, f, h and 8b, c, e are provided as a Source Data file. The raw RNA sequencing data are deposited at the ArrayExpress database [https://www.ebi.ac.uk/arrayexpress/] under accession numbers E-MTAB-8024 (Fig. 2a, b), E-MTAB-8025 (Fig. 2d, e) and E-MTAB-8028 (Fig 5a, b, c).

Field-specific reporting

Please select the one below that is the best fit for your research. If you are not sure, read the appropriate sections before making your selection.

☒ Life sciences ☐ Behavioural & social sciences ☐ Ecological, evolutionary & environmental sciences

For a reference copy of the document with all sections, see nature.com/documents/nr-reporting-summary-flat.pdf

Life sciences study design

All studies must disclose on these points even when the disclosure is negative.

Sample size	No statistical methods were used to predetermine sample size. Sample size were not calculated prior to performing experiment. To minimized the number of mice models used, sample size were determined to be sufficient to detect differences between groups. All comparisons had minimum of 3 data points. Non of the data points used was generated as technical replicate.
Data exclusions	The only data excluded from the manuscript is from the RNAseq and single-cell RNAseq experiments. In the case of RNAseq, genes that were not expressed in at least two samples were discarded. Cells in single-cell RNAseq data sets were excluded according to the quality control used in Seurat R Package. The exclusion criteria were pre-established by Seurat R Package.
Replication	All data used in this study were replicated (minum 2 independent experiments were used). All attempts for replication were successful.
Randomization	Animals were selected and allocated for experiments randomly.
Blinding	Investigators were not blinded as non-subjective measures were used (i.e. frequencies, total numbers of cell populations, weights,..). Bioinformatics were blinded for RNAseq and single-cell RNAseq library preparation and analysis. Blinded investigators were used for counting the dendritic cells in medullary and cortical regions of the thymus in microscopic slices.

Reporting for specific materials, systems and methods

We require information from authors about some types of materials, experimental systems and methods used in many studies. Here, indicate whether each material, system or method listed is relevant to your study. If you are not sure if a list item applies to your research, read the appropriate section before selecting a response.

Materials & experimental systems

n/a	Involved in the study	
☐	☒	Antibodies
☐	☒	Eukaryotic cell lines
☒	☐	Palaeontology
☐	☒	Animals and other organisms
☒	☐	Human research participants
☒	☐	Clinical data

Methods

n/a	Involved in the study	
☒	☐	ChIP-seq
☐	☒	Flow cytometry
☒	☐	MRI-based neuroimaging

Antibodies

Antibodies used

Anti-Involucrin Rabbit Polyclonal BioLegend Poly19244 cat# 924401 1:500
Anti-mouse Aire-FITC eBioscience 5H12 cat# 53-5934-82 1:200
Anti-mouse CD11c-APC/Cy7 BioLegend N418 cat# 117324 1:200
Anti-mouse CD11c-Biotin eBioscience N418 cat# 13-0114-82 1:100
Anti-mouse CD14-APC eBioscience Sa2-8 cat# 17-0141-82 1:100
Anti-mouse CD14-FITC eBioscience Sa2-8 cat# 11-0141-82 1:100
Anti-mouse CD172a (SIRPα)-APC BioLegend P84 cat# 144014 1:200
Anti-mouse CD172a (SIRPα)-PE/Cy7 BioLegend P84 cat# 144008 1:150
Anti-mouse CD172a (Sirpα)-Purified BioLegend P84 cat# 144003 1:500
Anti-mouse CD182 (CXCR2)-PE BioLegend SA045E1 cat# 149609 1:100
Anti-mouse CD19-PerCP/Cy5.5 BioLegend 6D5 cat# 115533 1:100
Anti-mouse CD25-Biotion BioLegend PC61 cat# 102003 1:100
Anti-mouse CD25-PE/Cy7 BioLegend PC61 cat# 102016 1:150
Anti-mouse CD25-PerCP/Cy5.5 BioLegend 3C7 cat# 101911 1:150
Anti-mouse CD274 (PD-L1)-PE/Cy7 BioLegend 10F.9G2 cat# 124313 1:200
Anti-mouse CD275 (ICOSL)-PE BioLegend HK5.3 cat# 107405 1:200
Anti-mouse CD282 (TLR2)-Biotin BioLegend T2.5 cat# 121804 1:100
Anti-mouse CD284 (TLR4)-Biotin eBioscience UT41 cat# 13-9041-80 1:100
Anti-mouse CD289 (TLR9)-FITC eBioscience M9.D6 cat# 11-9093-82 1:100
Anti-mouse CD301b (Mgl2)-PE/Cy7 BioLegend URA-1 cat# 146807 1:200

Anti-mouse CD317 (PDCA-1)-PerCP/Cy5.5 BioLegend 927 cat# 127021 1:150
Anti-mouse CD326 (EpCAM)-APC eBioscience G8.8 cat# 17-5791-82 1:3000
Anti-mouse CD326 (EpCAM)-PE BioLegend G8.8 cat# 118205 1:3000
Anti-mouse CD326 (EpCAM)-PE/Cy7 BioLegend G8.8 cat# 118215 1:3000
Anti-mouse CD3ε-APC BioLegend 145-2C11 cat# 100311 1:100
Anti-mouse CD3ε-Biotin BioLegend 145-2C11 cat# 100304 1:100
Anti-mouse CD40-APC eBioscience 1C10 cat# 17-0401-81 1:200
Anti-mouse CD45.1-PerCP/Cy5.5 BioLegend A20 cat# 110727 1:150
Anti-mouse CD45.2-PE eBioscience 104 cat# 12-0454-81 1:150
Anti-mouse CD45-PerCP/Cy5.5 BioLegend 30-F11 cat# 103131 1:100
Anti-mouse CD45RB-Biotin In house - - 1:100
Anti-mouse CD4-FITC eBioscience GK1.5 cat# 11-0041-82 1:200
Anti-mouse CD73-PB BioLegend TY/11.8 cat# 127211 1:200
Anti-mouse CD80-PerCP/Cy5.5 BioLegend 16-10A1 cat# 104721 1:200
Anti-mouse CD86-APC/Cy7 BioLegend GL-1 cat# 105029 1:100
Anti-mouse CD8a-PE BioLegend 53-6.7 cat# 100707 1:300
Anti-mouse CX3CR1-BV421 BioLegend SA011F11 cat# 149023 1:200
Anti-mouse I-A/I-E-FITC BD Pharmigen 2G9 cat# 553623 1:400
Anti-mouse I-A/I-E-PerCP/Cy5.5 BioLegend M5/114.15.2 cat# 107625 1:500
Anti-mouse Keratin 14-Purified BioLegend Poly19053 cat# 905303 1:500
Anti-mouse Ly-51-AF647 BioLegend 6C3 cat# 108311 1:200
Anti-mouse Ly-51-PE/Cy7 BioLegend 6C3 cat# 108313 1:200
Anti-mouse Ly-6G/Ly-6C (Gr-1)-APC BioLegend RB6-8C5 cat# 108411 1:400
Anti-mouse Ly-6G/Ly-6C (Gr-1)-PB BioLegend RB6-8C5 cat# 108429 1:400
Anti-mouse Ly-6G/Ly-6C (Gr-1)-PerCP/Cy5.5 BioLegend RB6-8C5 cat# 108427 1:400
Anti-mouse Siglec H-APC BioLegend 551 cat# 129611 1:100
Anti-mouse TCR Vα2-PE BioLegend B20.1 cat# 127808 1:100
Anti-mouse TCRβ-APC/Cy7 BioLegend H57-597 cat# 109220 1:150
Anti-mouse TCRβ-Biotin BioLegend H57-597 cat# 109204 1:100
Anti-mouse TER-119-Biotin BioLegend TER-119 cat# 116204 1:600
Anti-mouse TLR3-APC BioLegend 11F8 cat# 141905 1:200
Anti-mouse TLR5-AF647 BioLegend ACT5 cat# 148103 1:50
Anti-mouse Vβ5.1/5.2 TCR-FITC BD Pharmigen MR9-4 cat# 553189 1:50
Anti-mouse/human CD45R/B220-AF488 BioLegend RA3-6B2 cat# 103205 1:200
Anti-mouse/human CD45R/B220-APC BioLegend RA3-6B2 cat# 103211 1:200
Anti-mouse/human KLGR1-BV510 BioLegend 2F1/KLKGR1 cat# 138421 1:200
Anti-mouse/rat Foxp3-APC eBioscience FJK-16s cat# 17-5773-82 1:200
Anti-mouse/rat Foxp3-PE eBioscience FJK-16s cat# 12-5773-80 1:200
Anti-mouse/rat XCR1-APC BioLegend ZET cat# 148205 1:200
Anti-mouse/rat XCR1-BV421 BioLegend ZET cat# 148216 1:200
Cell proliferation Dye-eFluor 670 Invitrogen - cat# 65-0840-85 1:500
Fixable Viability Dye-eFluor 450 eBioscience - cat# 65-0863-14 1:300
Fixable Viability Dye-eFluor 506 eBioscience - cat# 65-0866-14 1:300
Goat anti rabbit-AF488 Invitrogen - cat# A27034 1:1000
Goat anti rabbit-AF647 Life Technologies - cat# A21244 1:1000
Goat anti rat-AF568 Life Technologies - cat# A11077 1:1000
Isotype control mouse-APC BioLegend RTK4174 cat# 400713 1:50/1:200
Isotype control mouse-FITC eBioscience eBR2a cat# 11-4321-80 1:100
Streptavidin-APC eBioscience - cat# 17-4317-82 1:500
Streptavidin-APC/Cy7 BioLegend - cat# 405208 1:500
Streptavidin-FITC eBioscience - cat# 11-4317-87 1:500
Streptavidin-PE/Cy7 BioLegend - cat# 405206 1:500

Validation

Most of the antibodies used are commercially available, and were validated by the manufacturer. Antibodies were tested in the laboratory using on known positive and negative controls and titrated. The Anti-mouse CD45RB-Biotin was generated in house. The function and specificity of this antibody was tested using positive and negative controls. FMO or a particular Isotype control staining was used to determine the staining positivity.

Eukaryotic cell lines

Policy information about cell lines

Cell line source(s)

A5 (NFAT-GFP) T-cell hybridoma cell line (Aschenbrenner et al. Nature Immunology 2007) were a gift from prof. Ludger Klein.

Authentication

None of the cell lines used were authenticated.

Mycoplasma contamination

All cell lines were tested for Mycoplasma by PCR on a regular basis. All cell lines were negative for Mycoplasma contamination.

Commonly misidentified lines (See ICLAC register) | No commonly misidentified cell lines were used in this study.

Animals and other organisms

Policy information about studies involving animals; ARRIVE guidelines recommended for reporting animal research

Laboratory animals

A majority of the mice used in this study were of C57BL/6J genetic background and housed in the animal facility at the Institute of Molecular Genetics of the ASCR v.v.i. under SPF conditions. Aire–/– (B6.129S2-Airetm1.1Doi/J, stock# 004743)2, Foxn1Cre (B6(Cg)-Foxn1tm3(cre)Nrm/J, stock# 018448)29, MyD88fl/fl (B6.129P2(SJL)-Myd88tm1Defr/J, stock# 008888), MyD88–/– (B6.129P2(SJL)-Myd88tm1.1Defr/J, , stock# 009088)30, Rag1–/– (B6.129S7-Rag1tm1Mom/J, stock# 002216)65, Ly5.1 (B6.SJL-PtprcaPepcb/BoyJ, stock# 002014)66, Cxcr2fl/fl (C57BL/6-Cxcr2tm1Rmra/J, stock# 024638)67, H2-Ab1fl/fl (B6.129X1-H2-Ab1tm1Koni/J, stock# 013181)68, and ItgaxCre (B6.Cg-Tg(Itgax-cre)1-1Reiz/J, stock# 008068)69 mice were purchased from Jackson Laboratories. Rosa26TdTOMATO (B6;129S6-Gt(ROSA)26Sortm14(CAG-tdTomato)Hze/J, stock# 007908)70 and Vav1Cre (B6.Cg-Commd10Tg(Vav1-icre)A2Kio/J, stock# 008610)71 were kindly provided by V. Kořínek (Institute of Molecular Genetics of the ASCR, Prague, Czech Republic). Aire-HCO (Balb/c)4 were provided by L. Klein. Cd3ε–/–72, RIP-OVA73, OT-I+Rag2–/–74 (all C57BL/6J) were provided by O. Štěpánek. OT-II (B6.Cg-Tg(TcraTcrb)425Cbn/J, stock# 004194)75 mice were kindly provided by T. Brdička (Institute of Molecular Genetics of the ASCR, Prague, Czech Republic). C57BL/6J GF and control C57BL/6J SPF mice were kindly provided by M. Schwarzer (Institute of Microbiology of the ASCR, Nový Hrádek, Czech Republic. Both GF and SPF mice were subject to the SSNIFF V1124-300 diet. Thymic cell populations were isolated from 3-6 week old mice with the exception of newborn mice (4 days old) used in Supplementary Fig. 7b. For the purpose of BM chimera experiments, 5-6 week old mice were irradiated and analysed between 11-13 weeks of age. Comparative analysis used age-matched cohorts regardless of sex and caging. Where possible, littermates were used as the controls. For the purpose of tissue isolation, mice were euthanized by cervical dislocation.

Wild animals

No wild animals were used in this study.

Field-collected samples

The study did not involve samples collected from the field.

Ethics oversight

All experiments were approved by the ethical committee of the Institute of Molecular Genetics and by the ethical committee of the Czech Academy of Science.

Note that full information on the approval of the study protocol must also be provided in the manuscript.

Flow Cytometry

Plots

Confirm that:

☐ The axis labels state the marker and fluorochrome used (e.g. CD4-FITC).

☐ The axis scales are clearly visible. Include numbers along axes only for bottom left plot of group (a 'group' is an analysis of identical markers).

☐ All plots are contour plots with outliers or pseudocolor plots.

☒ A numerical value for number of cells or percentage (with statistics) is provided.

Methodology

Sample preparation

Thymic antigen presenting cells, TECs and DCs, were isolated as follows. Thymus was minced into small pieces and treated with Dispase II (Gibco), dissolved in RPMI at concentration 0.1 mg.ml-1. Tissue was homogenized by pipetting and after 10 minutes of incubation (37°C), the supernatant was collected and the reaction was stopped by adding 3% FSC and 2nM EDTA. The process was repeated until all thymic fragments were digested. For thymic epithelial cells isolation, the whole thymic cell suspension was depleted of CD45+ cells by CD45 microbeads staining (Miltenyi biotec). Thymic dendritic cells were isolated using MACS enrichment for CD11c+ cells through staining with biotinylated CD11c antibody, followed by Ultrapure Anti-Biotin microbeads staining (Miltenyi biotec). For isolation of T-cell, thymus, peripheral lymph nodes (pLN), mesenteric lymph nodes (mLN) or spleen were mechanically mashed through 40μm Cell strainer (Biologix) and cell suspensions were passed through 50μm filters (Sysmex). The resulting cell suspension was spun down (4°C, 400g, 10 minutes) and erythrocytes were removed using ACK lysis buffer.

Instrument

LSRII flow cytometer (BD Biosciences), Influx cell sorter (BD Biosciences), AMNIS ImageStream X MkII

Software

BD FACSDIVA™ SOFTWARE V6.0 for LSRII (with HTS option), FlowJo V10 (10.5.3), IDEAS 6.1 software

Cell population abundance

When possible, the post-sorting analysis was performed, with average cell purity above 98%. For RNA isolation, cells were sorted directly to RNA lysis buffer.

Gating strategy

Gating strategies for individual experiments are depicted in respective Figures or Supplementary Figures. Boundaries between positive and negative populations were determined based on unstained, FMO or isotype controls.

☒ Tick this box to confirm that a figure exemplifying the gating strategy is provided in the Supplementary Information.
