# Supplementary material for: Toll-like receptor signaling in thymic epithelium controls monocyte-derived dendritic cell recruitment and Treg generation
Source: Nat Commun. 2020 May 12;11:2361. doi: 10.1038/s41467-020-16081-3 (PMC7217920; doi:10.1038/s41467-020-16081-3)
Supplement: Supplementary file 4 — Description of Additional Supplementary Files [file 41467_2020_16081_MOESM4_ESM.pdf]

Description of Additional Supplementary Files

Title: Supplementary Data 1

Description: (Related to Fig. 2b). A list of MyD88 induced genes in mTECs^{high}. RNA-sequencing data from mTECs^{high} derived from MyD88$^{fl/fl}$ and MyD88$^{\Delta TECs}$ mice. Data represents the analysis of n=5 samples for each condition. Statistical analysis was performed by Wald test, Fold-change cutoff of log2=+/-1,0 and p-value 0.05. Differentially expressed genes of interest are depicted in red.

Title: Supplementary Data 2

Description: (Related to Fig. 2b). A list of MyD88 repressed genes in mTECs^{high}. RNA-sequencing data from mTECs^{high} derived from MyD88$^{fl/fl}$ and MyD88$^{\Delta TECs}$ mice. Data represents the analysis of n=5 samples for each condition. Statistical analysis was performed by Wald test, Fold-change cutoff of log2=+/-1,0 and p-value 0.05.

Title: Supplementary Data 3

Description: (Related to Fig. 2d, e). A list of CpG ODN upregulated genes in mTECs^{high}. RNA-sequencing data from CpG ODN or PBS *in vitro* stimulated mTECs^{high} (sorted as in Supplementary Fig. 1a) from MyD88$^{+/+}$ and MyD88$^{-/-}$ mice (*n*=4 samples for each condition). Statistical analysis was performed by Wald test, Fold-change cutoff of log2=+/-1,0 and p-value 0.05. Differentially expressed genes of interest are depicted in red.

Title: Supplementary Data 4

Description: (Related to Fig. 2d, e). A list of CpG ODN downregulated genes in mTECs^{high}. RNA-sequencing data from CpG ODN or PBS *in vitro* stimulated mTECs^{high} (sorted as in Supplementary Fig. 1a) from MyD88$^{+/+}$ and MyD88$^{-/-}$ mice (*n*=4 samples for each condition). Statistical analysis was performed by Wald test, Fold-change cutoff of log2=+/-1,0 and p-value 0.05.

Title: Supplementary Data 5

Description: (Related to Fig. 5a, b, c). Differentially expressed genes determining DC-clusters. ddSEQ single-cell RNA-sequencing from FACS sorted Gr-1^{-}CD11c^{+}TdTOM^{+} DCs from the thymus of Foxn1^{Cre}ROSA26^{TdTOMATO} mice. Statistical analysis was performed by Wald test, Fold-change cutoff of log2=+/-1,0 and p-value 0.05.

Title: Supplementary Data 6

Description: (Related to Methods section). A full list of antibodies and primers used throughout the study.
